# Out-of-Sample Extrapolation utilizing Semi-Supervised Manifold Learning (OSE-SSL): Content Based Image Retrieval for Histopathology Images

**DOI:** 10.1038/srep27306

**Published:** 2016-06-06

**Authors:** Rachel Sparks, Anant Madabhushi

**Affiliations:** 1 University College of London, Centre for Medical Image Computing, London, UK; 2 Case Western Reserve University, Department of Biomedical Engineering, Cleveland, OH, USA

## Abstract

Content-based image retrieval (CBIR) retrieves database images most similar to the query image by (1) extracting quantitative image descriptors and (2) calculating similarity between database and query image descriptors. Recently, manifold learning (ML) has been used to perform CBIR in a low dimensional representation of the high dimensional image descriptor space to avoid the curse of dimensionality. ML schemes are computationally expensive, requiring an eigenvalue decomposition (EVD) for every new query image to learn its low dimensional representation. We present out-of-sample extrapolation utilizing semi-supervised ML (OSE-SSL) to learn the low dimensional representation without recomputing the EVD for each query image. OSE-SSL incorporates semantic information, partial class label, into a ML scheme such that the low dimensional representation co-localizes semantically similar images. In the context of prostate histopathology, gland morphology is an integral component of the Gleason score which enables discrimination between prostate cancer aggressiveness. Images are represented by shape features extracted from the prostate gland. CBIR with OSE-SSL for prostate histology obtained from 58 patient studies, yielded an area under the precision recall curve (AUPRC) of 0.53 ± 0.03 comparatively a CBIR with Principal Component Analysis (PCA) to learn a low dimensional space yielded an AUPRC of 0.44 ± 0.01.

Manual examination of prostate histopathology by an expert pathologist is the current gold standard for prostate cancer diagnosis, with roughly 242,000 new cases ever year^1^. The most common system of grading prostate cancer is the Gleason score^2^, determined as a summation of the two most prevalent Gleason patterns. Low Gleason grade patterns (1–3) are reflective of less aggressive disease while high Gleason grade patterns (4–5) are reflective of more aggressive disease. The most dominant Gleason patterns, comprising around 90% of needle biopsies cases, are patterns 3 and 4^3^. Correctly distinguishing between primary Gleason patterns 3 and 4 is critical for determining the appropriate treatment for patients; patients with less aggressive disease (primary Gleason grade patterns ≤3) are enrolled in active surveillance programs while patients with more aggressive disease (primary Gleason grade patterns ≥4) undergo treatment^4^. Additionally, distinguishing between the intermediate Gleason patterns 3 and 4 is a particularly challenging task, with inter-observer agreement between pathologists as low as 0.47–0.64 (reflecting low to moderate agreement). Hence a method to consistently distinguish between these patterns is an important clinical need^5^.

Prostate glands are considered an important substructure when assessing Gleason grade^2^, and gland morphology has been shown to discriminate between benign and malignant tissue regions^6^,^7^,^8^. A content-based image retrieval (CBIR) system which can accurately retrieve prostate histopathology according to Gleason grade pattern can be useful in a clinical, research and educational setting. To enable ease of reading Table 1 lists common acronyms used throughout this manuscript.

An accurate CBIR system for retrieval of Gleason grade patterns can aid in the training of medical students and could allow pathology residents to hone in on their Gleason grading skills^6^,^7^,^9^. In this work we present Out-of-Sample Extrapolation utilizing Semi-Supervised Learning (OSE-SSL) for CBIR of prostate histopathology. OSE-SSL allows for CBIR of the prostate histopathology images to be performed in an accurate and computationally efficient manner. To determine image similarity we leverage our previously developed method, Explicit Shape Descriptors (ESDs)^8^, to distinguish between prostate glands from different Gleason grade patterns. ESDs involve fitting an explicit medial axis shape model to each gland of interest, computing pairwise differences between shape models, and then extracting a set of feature via manifold learning (ML). ESDs have been shown to have over 80% classification accuracy in distinguishing prostate glands belonging to Gleason grade patterns 3 and 4^8^. This current work is distinct from^8^ due to the following reasons: (1) in this paper we present a computationally efficient method (OSE-SSL) for retrieving images in a low dimensional representation of the feature space, while in^8^ the methodology for ESDs extraction was presented; (2) in this paper we focus on evaluating OSE-SSL in terms of computational efficiency and precision-recall accuracy, while in^8^ we focused specifically on the classification accuracy of ESDs; and (3) OSE-SSL is a method that can be used in conjunction with any set of image features, while in^8^ ESDs are a specific method of extracting morphologic features from an object of interest.

CBIR systems attempt to retrieve images from a database identified as being the most similar to the query image in terms of quantitative image descriptors obtained from the query and database images. In the context of medical imagery, images which are visually similar often have similar pathologies. A CBIR system for histopathology images could serve as a useful training tool for pathology residents, fellows, and medical students and could potentially serve as a decision-support tool in diagnosis and grading of pathologies^6^,^7^,^9^,^10^,^11^,^12^,^13^,^14^,^15^,^16^. CBIR systems are particularly relevant in the context of histopathology imagery where (a) the images can be extremely large and described by a very large set of image descriptors, and (b) differences between pathologies may be very subtle and not immediately appreciable visually. Additionally, with the recent advent of whole-slide digital scanners, pathology labs will soon be routinely generating very large amounts of digitized histopathology imagery, necessitating intelligent and efficient image retrieval systems^17^.

CBIR systems typically comprise two components: (1) a module for extraction of domain specific image descriptors to quantitatively characterize the images, and (2) a module for computation of the similarity between the query and database images in terms of the quantitative image descriptors. Histopathology images typically comprise several billions worth of pixels^17^, and hence histopathology CBIR systems require a large number of image descriptors to accurately describe subtle differences in the complex imagery^6^,^7^,^9^,^10^,^12^,^13^,^14^,^15^,^16^. Such medical imagery can be represented by a high dimensional space, where each dimension corresponds to a single image descriptor. A high dimensional image descriptor space makes the calculation of similarity between image descriptors difficult as (a) the number of database images may be small compared to the number of image descriptors giving rise to the curse of dimensionality problem^18^, and (b) images often cluster densely in small regions of the high dimensional space^19^. Hence relationships between image descriptors may be important when calculating image similarity. Consequently, a few researchers have proposed dimensionality reduction (DR) methods^6^,^7^,^9^,^10^,^16^,^20^ to map the high dimensional image descriptors into a low dimensional representation so that image similarity calculation and retrieval can be performed directly in the low dimensional space. Retrieval performed in a low dimensional space is often more accurate than retrieval performed in the original high dimensional space^6^,^7^. However, utilizing DR methods to learn a low dimensional space may add computational complexity to the retrieval algorithm.

Linear DR methods, such as Principal Component Analysis (PCA), attempt to find a low dimensional space that is a linear projection of the high dimensional space. Hence linear DR methods only preserve linear relationships between images^9^,^10^. Semi-supervised learning (SSL) methods, such as Linear Discriminant Analysis (LDA), have been proposed to take into account semantic information such as partial class labels when learning a low dimensional projection in order to co-localize semantically similar images^13^,^21^,^22^. However, these methods assume that a linear projection of the high dimensional space will best preserve relationships between images. ML schemes attempt to find a low dimensional embedding space which preserves the manifold structure of the image descriptors in the high dimensional space. Hence ML methods attempt to preserve the non-linear relationships between image descriptors^23^,^24^,^25^. Graph Embedding (GE)^23^, a specific instance of a ML scheme, attempts to model the manifold structure using local, pairwise relationships between image descriptors in the high dimensional space thereby preserving these relationships between images in the low dimensional space. Recent work has demonstrated that ML schemes, such as GE, may result in low dimensional spaces better suited for CBIR when image similarity is defined by a non-linear manifold in the high dimensional space^6^,^7^,^16^,^20^. Semi-supervised ML methods, which utilize SSL in conjunction with ML, attempt to learn a low dimensional embedding space such that semantic, non-linear relationships between images in the high dimensional space are preserved^26^. To our knowledge no CBIR systems for histopathology have leveraged SSL. However, CBIR systems for color photography^20^,^27^ have been proposed which leverage such methods.

Figure 1 demonstrates the ability of GE to preserve non-linear relationships between samples for the synthetic Swiss Roll dataset. Figure 1(a) shows a synthetic Swiss Roll dataset consisting of 2000 samples described by a 3*D* space, the arrow demonstrating the direction of greatest variance along the manifold. In this example GE is able to find a low dimensional space (2*D*) which preserves the underlying structure of the dataset as evidenced by the planar 2*D* embedding space shown in Fig. 1(b). Figure 1(c) shows the results of semi-supervised GE (SSGE) for the Swiss Roll. Note that for SSGE (Fig. 1(c)) samples from two classes (blue, red) have a larger separation compared to GE (Fig. 1(b)).

Despite the advantages of ML, only a few papers have attempted to use ML in conjunction with CBIR of medical imagery^6^,^7^,^16^, due to its computational cost. A computationally expensive eigenvalue decomposition (EVD) must be calculated for every new query image^28^,^29^. Hence there is a need to develop ML schemes which are more computationally efficient and do not require a EVD for each new query image. Algorithms have been developed to avoid recomputing the EVD for out-of-sample images, but have not previously been evaluated in the context of CBIR for medical imagery^28^,^29^.

Locality Preserving Projections (LPP) attempts to approximate the low dimensional embedding space found by ML as a linear combination of image descriptors in the high dimensional space^28^. LPP is reliant on a linear combination of the image descriptors accurately modeling relationships between images, and hence accurately modeling relationships in the low dimensional space. If the low dimensional space found via ML is not approximately linear LPP will not correctly estimate the low dimensional space. Alternatively, out-of-sample extrapolation (OSE)^29^ attempts to determine the location (or embedding) of a new query image in the low dimensional embedding space as a weighted sum of the embeddings already calculated for a set of images. In the context of a CBIR system, the calculated embeddings would correspond to the locations for the database images. Unlike LPP, non-linear relationships between images are preserved and, hence, OSE may be better able to resolve differences between images belonging to different classes. Figure 1(d) shows the result of OSE for the Swiss Roll dataset where samples projected into the low dimensional space via OSE are represented by open points.

In this paper we present OSE-SSL a novel and unique combination of SSL^13^,^21^,^22^ and OSE^29^ into a unified framework. The OSE-SSL algorithm represents a novel, non-obvious combination of these two popular methods. The OSE-SSL algorithm first refines relationships between images in the low dimensional embedding space according to semantic information via SSL and then utilizes OSE to project never before seen images into the low dimensional space learned via SSL.

## Previous work and novel contributions

Several CBIR methods for radiological medical imagery have been presented^21^,^30^. Such CBIR systems extract relatively few image descriptors and hence are able to accurately perform image retrieval in the original high dimensional image descriptor space. In comparison, CBIR systems for histopathology imagery extract a very large number of features to describe the complex imagery^6^,^7^,^9^,^10^,^12^,^13^,^14^,^15^,^16^,^31^,^32^,^33^,^34^,^35^,^36^ and therefore typically perform image retrieval in a reduced dimensional space to overcome the curse of dimensionality problem.

Comaniciu *et al*.^10^ utilized a weighted sum of image descriptors, where weights were determined by maximizing an objective function, to retrieve images corresponding to different hemotologic malignancies. This approach is equivalent to a linear DR method as only linear relationships between images are preserved during retrieval. Yang *et al*.^15^ utilized a similar approach on a larger database of hemotologic malignancies. Similarly Zhang *et al*.^32^ determined a weighted sum of image descriptors using a Pareto archived evolution strategy to learn the best weights for their image retrieval task. Zheng *et al*.^9^ utilized multi-dimensional scaling (MDS), a linear DR scheme, to compute a low dimensional space in which image retrieval could be performed for a set of histopathology images taken from different anatomical regions (e.g. spleen, prostate, colon, etc.).

Tang *et al*.^12^ obtained different low dimensional spaces by considering different image descriptors, to obtain a corresponding set of semantic labels. Image retrieval of colon histopathology images was then performed by returning images with the most similar semantic labels across the different low dimensional spaces. Yu *et al*.^14^ took a similar approach but introduced spatial constraints to determine the semantic labels and image similarity for colon histopathology images. Caicedo *et al*.^31^ used a non-negative matrix factorization to determine a mapping between image features and semantic terms; new images could be projected into the learned feature space to retrieve similar images.

Lessmann *et al*.^13^ used self organizing maps to determine the most important image descriptors for meningioma histopathology; the top 6 image descriptors were selected to determine image similarity. Caicedo *et al*.^16^ determined similarity between basal-cell carcinoma histopathology images by learning a set of kernels and associated weights for each image descriptors. Such a scheme is equivalent to ML, as non-linear relationships between image descriptors are taken into account. This method required an extensive offline training phase to learn the kernels and weights utilized in the similarity measure.

Zhang *et al*.^33^,^35^ use a semi-supervised hashing method in combination with a set of kernels to learn a non-linear feature space that can quickly and efficiently describe feature similarity between images using SIFT features. Zhang *et al*.^36^ applied a semi supervised hashing method to cell-based features for image retrieval of histpathology images. Jiang *et al*.^34^ used a similar hashing method, but used a joint kernel representation for both image features and labels to learn a hash representation that could better model class relationships between images.

Previous work from our group used GE to find a low dimensional embedding space and then retrieved prostate histopathology images according to image similarity in the low dimensional space^6^. This method was able to take into account non-linear relationships between image samples without an extensive offline learning phase. However, GE must recalculate a computationally expensive EVD for every out-of-sample image, or every query image not contained in the database images.

**Figure 2:**
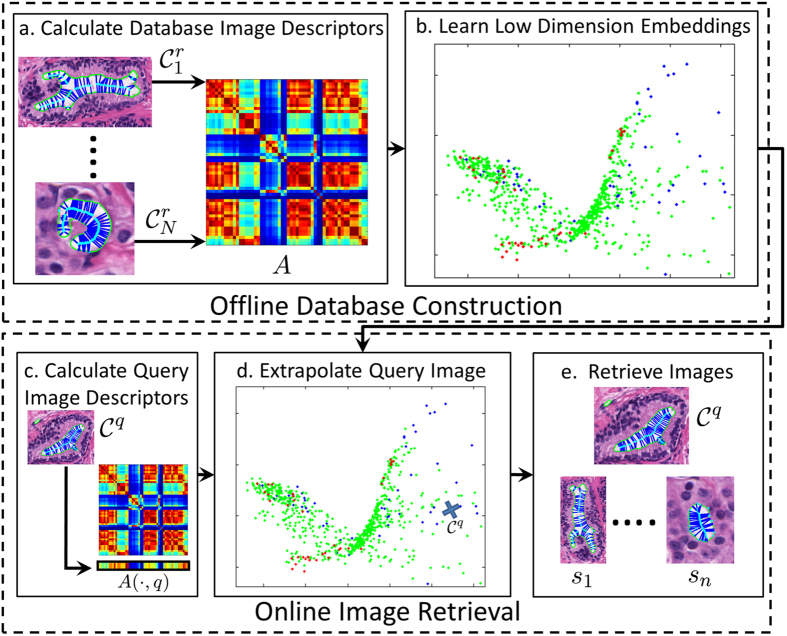
A flowchart of the OSE-SSL CBIR system. The system has an offline database construction phase (top) and an online retrieval phase (bottom). Database construction consists of (**a**) obtaining a set of *N* repository images 
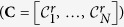
 and extract image features, represented by the dissimilarity matrix *A*. (**b**) Performing SSL to learn the low dimensional embedding space which optimally describes similarity between images in **C**. Retrieval of images most similar to a query image 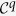 is then performed via (**c**) extracting image features from 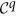, represented by *A*(·, *q*). (**d**) OSE of 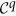 into the low dimensional embedding space. (**e**) Image retrieval of the *n* most similar images (*s*_1_, …, *s*_*n*_) to 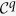 according to Euclidean distance in the low dimensional embedding space.

In this work we have developed OSE-SSL algorithm, which represents a novel combination of SSL and OSE, designed specifically to be computationally tractable. The novel integration of these two methods involves projecting never-before seen images into a low dimensional embedding space that takes into account semantic information (class label information). Hence OSE-SSL (a) integrates known label information to learn a low dimensional embedding space and (b) overcomes the out-of-sample problem. We demonstrate the use of OSE-SSL in the context of CBIR applications. Figure 2 illustrates a flowchart of our OSE-SSL CBIR system. The CBIR system is characterized by (1) offline database construction where SSL is applied to quantitative image descriptors for a set of database images to obtain a low dimensional embedding space and (2) online image retrieval where OSE is used to compute the embedding location of a never before seen query image. Offline database construction consists of (a) extracting image descriptors for all database images, and (b) applying SSL to determine the low dimensional embedding space for images contained within the database. Once offline database construction has been completed online image retrieval is then performed efficiently utilizing OSE. Online image retrieval consists of (c) extracting image descriptors from the query image, (d) OSE of the query image into the low dimensional embedding space, and (e) ranking image similarity in the low dimensional embedding space.

The novelty of OSE-SSL is four-fold: (1) OSE-SSL leverages partial class label information, where available, when learning the low dimensional space by utilizing SSL, (2) OSE-SSL extrapolates a new query image into the low dimensional space without re-computing an EVD by utilizing OSE, making OSE-SSL computationally tractable compared to SSL and hence, ideally suited to CBIR applications, (3) OSE-SSL represents a novel, non-obvious combination of OSE and SSL into a single unified framework, and (4) OSE-SSL is demonstrated in the context of a novel application of CBIR to the Gleason grading problem.

In this work, we demonstrate the application of OSE-SSL to a CBIR system which retrieves images according to morphologic similarity. Morphologic similarity is determined via ESDs, a morphologic descriptor which is determined by (1) fitting a medial axis shape model to each shape, (2) determining pairwise similarity between images, and (3) performing non-linear dimensionality reduction on the pairwise similarity^8^.

We evaluate our system on two datasets a digitized prostate histopathology dataset. The prostate histopathology dataset was chosen due to the challenges in accurately distinguishing between intermediate Gleason grade patterns^5^; ESDs have been shown to have over 80% classification accuracy in distinguishing between prostate glands from Gleason grade pattern 3 and Gleason grade pattern 4 as seen on histopathology^8^. Hence a CBIR system which leverages ESDs should be able to accurately retrieve histopathology images according to Gleason grade pattern.

## Out-of-Sample Extrapolation Utilizing Semi-Supervised Manifold Learning (OSE-SSL)

### Notation

Table 2 displays the notation used in the paper. A database of *N* images is defined by 
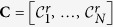
. *r* denotes that the image is contained in **C**, to contrast with 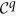 where *q* denotes a query image not contained in database. Each image in the database has a corresponding label defined by **L** = [*l*_1_, …, *l*_*N*_]. Every label *l*_*i*_ ∈ **L** takes on a discrete value *l*_*i*_ ∈ {1, 2, …, *Z*} where **C** contains images belonging to *Z* classes.

For two images 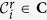 and 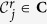, *j* ≠ *i* we define pairwise dissimilarity as 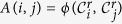. The function *ϕ*(·, ·) can represent any dissimilarity function such that if 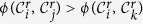 then it follows that 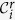 and 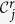 are more dissimilar than 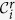 and 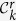. The function 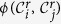 is evaluated over all *i, j* ∈ {1, …, *N*}, *j* ≠ *i* to obtain *A. A* is an *N* × *N* matrix representing pairwise dissimilarity between all images contained in **C**.

### Review of Manifold Learning

#### Graph Embedding

The goal of GE is to determine a set of low dimensional embedding locations 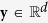 that preserves the relationships between images in 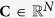 where 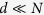. GE determines **y** by modeling the similarity between images according to a similarity matrix *W*. Given the dissimilarity matrix *A* described in the Notation Section, *W* is found by *W*(*i, j*) = *e*^−*A*(*i, j*)/*σ*^, where *σ* is a user selected scaling parameter. **y** is then found by minimizing the pairwise reconstruction error defined as,


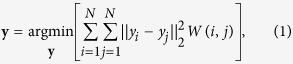


where ||·||_2_ denotes the L2-norm. An image 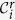 is associated with the embedding location *y*_*i*_. Belkin *et al*.^37^ demonstrated that Equation 1 is equivalent to the following eigenvalue decomposition (EVD),


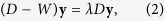


where *D* is a diagonal matrix defined as 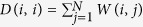. The smallest *d* eigenvalues, excluding any 0 valued eigenvalues, in *λ* correspond to the *d* eigenvectors **y** which are defined as the *d* dimensional embedding locations. **y** correspond to the projection of the matrix *W* into 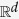 such that the pairwise similarity between the elements in *W*, and hence the pairwise similarity between images, are preserved. Furthermore the eigenvectors **y** are orthonormal, hence, each additional eigenvector (or dimension) provides independent information on the image similarity in *W*.

#### Semi-Supervised Manifold Learning (SSL)

For **C** let a corresponding set of known labels be defined as **L**_*r*_ ⊂ **L** where **L**^*r*^ = [*l*_1_, …, *l*_*M*_]. Note that *M* < *N* as we assume that some labels may be unknown for images contained in **C**. A similarity matrix *W*^*r*^ is constructed by altering elements in *W* according to **L**^*r*^. Images which correspond to the same class have higher values in *W*^*r*^ compared to *W*, while images which correspond to different classes have lower values in *W*^*r*^ compared to *W*. For images where no label information is known the values in *W*^*r*^ and *W* are equivalent. *W*^*r*^ is calculated as,


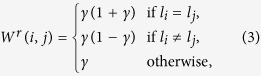


where *γ* = *W*(*i, j*). The “otherwise” case corresponds to instances where label information is unknown for either *l*_*i*_ or *l*_*j*_. Once the similarity matrix *W*^*r*^ has been calculated, the EVD described by Equation 2 is performed on *W*^*r*^ to obtain **y**^*r*^.

By altering *W*^*r*^ according to Equation 3, images belonging to the same class (i. e. *l*_*i*_ = *l*_*j*_) will be close together in the low dimensional embedding space. Images belong to different classes (i. e. *l*_*i*_ ≠ *l*_*j*_) will be farther apart in the low dimensional embedding space. Images where class information is unknown (i.e. *l*_*i*_ or *l*_*j*_ are undefined) will be near images determined to be similar, in terms of *ϕ*(·, ·), regardless of class.

#### Out-of-Sample Extrapolation (OSE)

OSE uses **y** determined from **C** to extrapolate *y*^*q*^ for 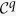. Assuming that **y** accurately describes the non-linear relationships in **C**, which should be the case when **C** is sufficiently large, OSE is able to accurately determine *y*^*q*^^38^,^39^.

OSE is divided into three steps,

1. Manifold Learning: A set of low dimensional embeddings **y** are learned by performing GE on **C** as described in the Graph Embedding Section.
2. Query Image Descriptor Calculation: Pairwise dissimilarity *A*(*i, q*) is calculated between 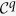 and every image contained in **C**. *W*(*i, q*) is calculated from *A*(*i, q*).
3. Query Sample Extrapolation: The embedding location *y*^*q*^ for 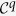 is extrapolated via,

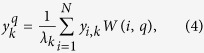


where *k* ∈ {1, …, *d*} is the *k*th embedding dimension corresponding to the *k*th smallest eigenvalue *λ*_*k*_.

Intuitively, OSE calculates *y*^*q*^ as a weighted sum of the database embeddings *y*_*i*_: *i* ∈ {1, …, *N*} where weights are based on image similarity described by *W*(*i, q*).

### Out-of-Sample Extrapolation for Semi-Supervised Manifold Learning

OSE-SSL is a novel combination of the previously described SSL and OSE algorithms that projects never-before seen images into a low dimensional embedding space that incorporates semantic information. OSE-SSL calculates **y**^*r*^ for **C** such that (a) the image class labels **L**^*r*^ are taken into account and (b) image similarity is optimally represented by **y**^*r*^. After **y**^*r*^ have been calculated for **C**, a new never before seen image 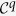 can be extrapolated into the low dimensional space to obtain **y**^*q*^. OSE-SSL calculates the embedding **y**^*q*^ in a computationally efficient manner.

Our novel methodology for OSE-SSL can be divided into an offline *ConstructOSE-SSL* algorithm and an online *ApplyOSE-SSL* algorithm both of which are described in detail below.

#### OSE-SSL Algorithm

The algorithm for OSE-SSL is divided into two parts, (1) *ConstructOSE-SSL* which is an offline computationally intensive algorithm to learn **y**^*r*^ that only needs to be performed once for **C** and (2) *ApplyOSE-SSL* which is an online algorithm to extrapolate *y*^*q*^ for 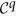. The combination of these two algorithms results in a low dimensional representation for both **C** and 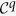.

The *ConstructOSE-SSL* algorithm takes into account only images contained in the database **C** and the corresponding semantic information **L**^*r*^. The algorithm is as follows,

**Algorithm** *ConstructOSE-SSL*

**Input**: **C**, **L**^*r*^

**Output**: *λ*^*r*^, **y**^*r*^

*begin*

1. Find 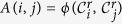 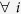, *j* ∈ {1, …, *N*}.
2. Find *W*^*r*^ by Equation 3.
3. Find *λ*^*r*^, **y**^*r*^ by Equation 2.

*end*

As with other SSL algorithms the use of labels **L**^*r*^ alters the similarity matrix *W*^*r*^ so that images belonging to the same class are more similar. The structure of *W*^*r*^ is changed such that *W*^*r*^ can be approximated as a block matrix where each block consists of samples belonging to the same class. An approximate block matrix formulation results in the EVD producing **y**^*r*^ that are more representative of the class differences that exist in the database **C**.

Once the eigenvalues *λ*^*r*^ and the embedding locations **y**^*r*^ have been computed, extrapolation of 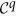 into the low dimensional embedding space can be performed via the *ApplyOSE-SSL* algorithm,

**Algorithm** *ApplyOSE-SSL*

**Input**: *C*^*q*^, *λ*^*r*^, **y**^*r*^

**Output**: *y*^*q*^

*begin*

1. Find 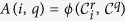 for all *i* ∈ {1, …, *N*}.
2. Calculate *W*(*i, q*) = *e*^−*A*(*i*,*q*)/*σ*^.
3. Find *y*^*q*^ by Equation 4.*end*

These two algorithms in combination allow for a low dimensional embedding space to be found for **C** and 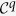. The use of **y**^*r*^ which are more reflective of the underlying class differences in **C** to calculate *y*^*q*^ provides two benefits. Firstly, *y*^*q*^ will capture more information pertaining to class differences in **C** when using **y**^*r*^ compared to the unsupervised embeddings **y**. Secondly, tthe error between *y*^*q*^ and its true location (determined by performing an EVD) will be smaller when calculated from **y**^*r*^ that contain relevant class differences in **C**.

#### Application to Image Retrieval

The goal of a CBIR system is to retrieve *b* images in **C** which are most similar to 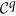. The application of OSE-SSL to a CBIR system can be applied to learn the metric 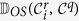 where 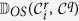 is defined such that smaller values correspond to more similar images.

Offline database construction is an important precursor to image retrieval and is performed using the algorithm *ConstructOSE-SSL*. Online retrieval of the most similar images in **C** is performed by the algorithm *RetrieveOSE-SSL* as follows,

**Algorithm** *RetrieveOSE-SSL*

**Input**: *C*^*q*^, **y**^*r*^

**Output**: 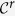


*begin*

1. Extrapolation of *y*^*q*^ for 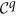 via *ApplyOSE-SSL*.
2. Calculation of similarity between **C** and 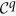 by, 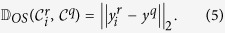
3. Sort 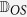 from smallest to largest value to give **s**.
4. Return 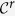 corresponding to the smallest *b* values in **s**.

*end*

#### OSE-SSL Computational Complexity

To analyze the computational complexity of our novel OSE-SSL algorithm we consider *ConstructOSE-SSL* and *ApplyOSE-SSL* separately. *ConstructOSE-SSL* is a SSL algorithm applied to **C**. SSL has a computational complexity of *O*(*N*^3^) due to the EVD in Equation 2 which is the rate limiting step^40^. However as *ConstructOSE-SSL* is utilized only to learn a low dimensional representation of **C** it is performed offline prior to image retrieval. *ApplyOSE-SSL* learns *y*^*q*^ for 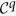 and hence must be performed online. The computational complexity of OSE is *O*(*N*) due to the weighted summation in Equation 4 40.

## Experimental Design and Results

We evaluated our *RetrieveOSE-SSL* algorithm on a prostate histpathology dataset described below. This dataset demonstrates the application of *RetrieveOSE-SSL* in retrieving images by Gleason grade using gland morphology. All code was implemented in MatLab^®^ 2012b and run on a computer with a 3.0 GHz Xeon Quad-Core processor and 16 GB of RAM.

### Prostate Histopathology Data Description

Prostate tissue biopsy cores were obtained from 58 patient studies. Each tissue biopsy was stained with Hemotoxylin and Eosin (H&E) and digitized using a ScanScope CS^TM^ whole-slide scanning system at 0.25 *μm* per pixel (40× optical magnification). An expert pathologist selected regions of interests (ROIs) on the digitized biopsy image, for a total of 102 ROIs. The expert pathologist then classified each ROI as benign (BE) (24 ROIs), Gleason grade 3 (G3) (67 ROIs), or Gleason grade 4 (G4) (11 ROIs). Every gland contained within each ROI was segmented by a human expert to obtain lumen and nuclear boundaries, the human expert was blinded to the Gleason grade for all glands. Glands which did not contain either a nuclear or lumen boundary, or where the contour was not fully contained within the ROI were removed from the study, resulting in a total of 888 glands. Glands were distributed across the three classes: BE (93), G3 (748), and G4 (47). Dissimilarity between prostate histopathology images is determined according to morphologic similarity between prostate glands on each image. The function 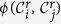 is calculated by leveraging ESDs, a method previously developed by our group^8^.

### Database Construction

For the dataset a query image 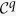 was selected such that each image in the dataset was selected once. **C** was constructed by randomly selecting *N* images, where *N* was empirically determined, from the dataset in such a way as to always maintain class balance. Class balance was maintained by always selecting the same ratio of each class of images, i.e. constructing **C** via stratified sampling of the dataset images. Additionally, the query image 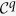 was always excluded from **C**. Construction of **L**^*r*^ was performed by randomly selecting *M* labels, where *M* was empirically determined, from the images in **C** in such a way as to maintain class balance. Additionally for all experiments *M* ≤ *N*, so that the total number of known labels were always less than or equal to *N*.

The OSE-SSL algorithm has two important empirically determined parameters, dataset size *N* and number of known labels *M*. To enable direct comparison between *N* and *M* these parameters are evaluated as a fraction of the dataset size. Specifically, we define a parameter *n* is a value between 0 and 1 such that *N* = *n* × *N*_*all*_. Hence *n* = 0.5 indicates that half of the total dataset available is being used to construct **C**. Similarly, *m* is defined to be a value between 0 and *l* such that *M* = *m* × *N*. Hence, *m* = 0.5 represents half of the labels in the database **C** being known.

### Evaluation Measures

OSE-SSL was evaluated on (a) Silhouette Index (SI) of **y**, a measure of how well images cluster according to class^41^, and (b) area under the precision-recall curve (AUPRC) of *RetrieveOSE-SSL*, a description of the behavior of an image retrieval system in terms of how many and in what order relevant images are returned. Table 3 describes all evaluation measures.

### Experiment 1: Distance Metric for Prostate Histopathology Database

In this experiment we evaluated the ability of 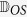 to retrieve relevant images for the prostate histopathology dataset. Five other distance metrics discussed in Table 4 were used for comparison. 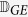 is a special case of 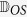 where 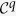 is contained in **C** (equivalent to *m* = 0.0 and *n* = 1.0), hence, *y*^*q*^ is calculated using Equation 2 for GE and is a non-linear unsupervised feature space. Distance metrics were chosen in order to evaluate the original feature space 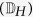, a linear unsupervised feature space 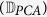, a linear approximation of an unsupervised non-linear feature space 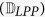, and a non-linear semi-supervised feature space that uses kernel-based hashing 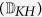. For 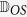 and 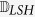 some labels are known (*m* = 0.5) and not all images are contained in the database (*n* = 0.9). For 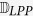 not all images are contained in the database (*n* = 0.9). The number of dimensions for 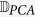, 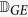, 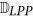, and 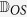 as well as the scaling parameter *σ* corresponding to the best retrieval performance were determined empirically and are reported in Table 5. Parameters for 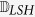 were determined as described in^42^.

**Figure 3:**
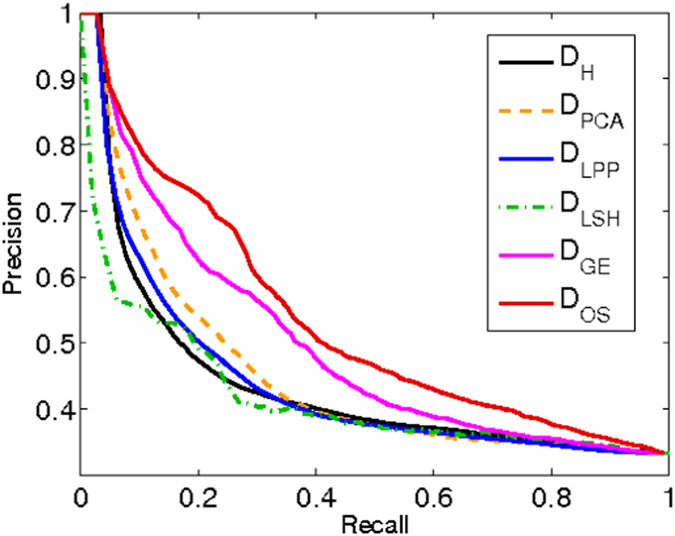
Precision-recall curves for Experiment 1 showing retrieval for the metrics: 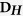 (black), 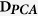 (orange), 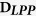 (blue), 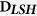 (dark green), 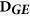 (pink),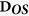 (dark red). The precision-recall curves for 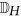, 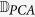, 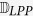 perform similarly. 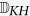 has a slightly worse performance than the other measures. 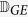 improve precision-recall, and 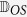 outperforms all other measures, as it leverages label and neighborhood information.

AUPRC and SI were calculated on a set of query images such that each image in the dataset was selected once. In Table 5 we report SI and AUPRC average value ± standard deviation over all 888 query images in the prostate histopathology databse for each distance metric. Figure 3 displays the AUPRC curves for each distance metric. 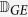 performs better retrieval, in terms of higher AUPRC and SI compared to either 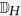 or 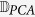. These differences were found to be statistically significant (*p* < 0.05) using a paired two-sided Student’s t-test where the null hypothesis was that the performance of 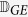 was not different compared to another distant metric (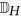, 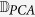, or 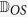). Additionally, increases in SI and AUPRC for 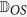
(*n* = 0.9, *m* = 0.5) compared to 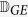 was found to be statistically significant.

**Figure 4:**
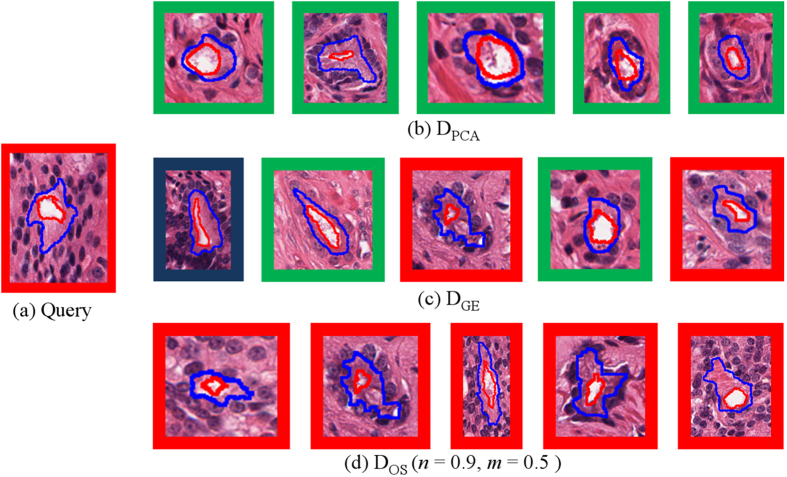
(**a**) G4 query image and top 5 images retrieved (left to right) by (**b**) 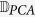, (**c**) 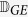, and (**d**) 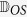. Retrieved images belonging to the same class as the query image (G4) are outlined in red while those belonging to G3 are in green, and BE are in blue.

Figure 4 displays the top 5 retrieved images for a G4 gland query image. 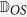
(*n* = 0.9, *m* = 0.5) was able to retrieve only glands belonging to the same class as 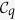. 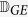 retrieved some glands incorrectly, probably due to the retrieved BE and G3 glands being atypical in shape and size for their class. The use of class information to learn the embeddings most likely allowed 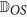 to learn a greater range of morphology traits for each class. 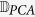 was unable to retrieve any glands belonging to the same class. The failure to retrieve G4 glands is most likely due to 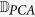 only capturing the main characteristics of the query gland, being small and roughly circular, while failing to capture the subtle undulations in the gland boundary that is a distinguishing feature between G3 and G4 glands.

**Figure 5:**
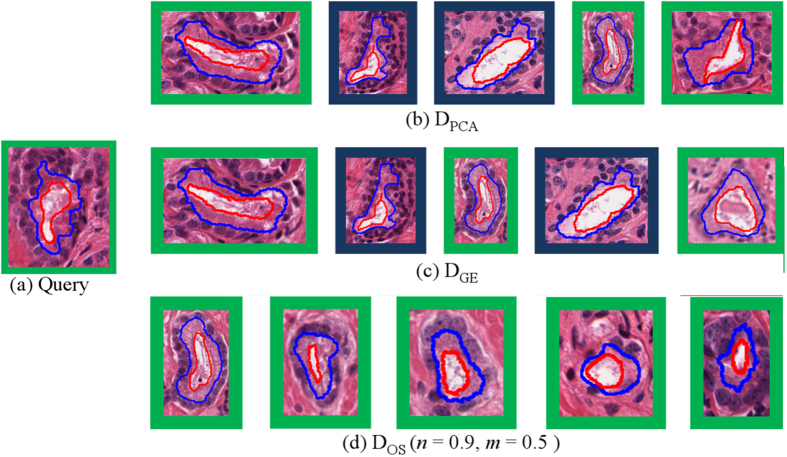
(**a**) G3 query image and top 5 images retrieved (left to right) by (**b**) 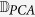, (**c**) 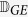, and (**d**) 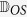. Retrieved images belonging to the same class as the query image are outlined in green (G3) while those belonging to BE are in blue, no G4 glands were retrieved.

Figure 5 shows the top 5 retrieved images for a G3 gland query image. 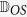
(*n* = 0.9, *m* = 0.5) was able to retrieve only glands belonging to the same class as 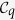. All of these images were of small, elongated glands. Both 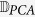 and 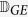 retrieved the same BE glands mistakenly in addition to G3 glands. The BE glands are elongated but slightly larger and having different patterns in terms of boundary perturbations.

**Figure 6:**
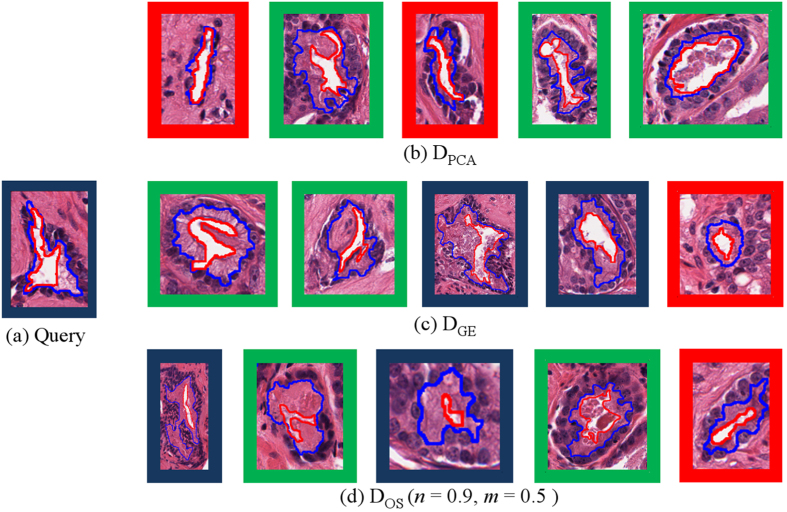
(**a**) BE query image and top 5 images retrieved (left to right) by (**b**) 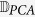, (**c**) 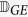, and (**d**) 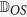. Retrieved images belonging to the same class as the query image are outlined in blue (BE) while those belonging to G3 are in green, and G4 are in red.

Figure 6 displays a particularly hard to classify 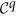 of a BE gland and the corresponding top 5 images retrieved. Further evaluation of this gland showed that due to its small size compared to other BE glands, Φ(·, ·) often resulted in a higher than expected dissimilarity between this gland and other BE glands, resulting in retrieving glands belonging to other classes. 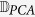 did not retrieve any glands belonging to the same class in this example. 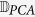 embeddings did note to capture subtle gland features, the gland size and the boundary undulations, that distinguish BE glands from other grades. 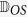 and 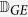 were able to retrieve glands belonging to the same class. However, 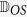 ranked glands belonging to the same class higher compared to 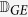. For both 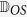 and 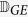, glands retrieved from different classes were likely to have subtle differences in the nuclear and lumen boundary attributes, cues that were not captured by any embedding space.

### Experiment 2: Parameter Sensitivity

In this experiment we evaluated the ability of 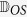 to retrieve relevant images for the prostate histopathology dataset under for a range of parameter conditions. For 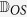 there are two parameters which may be selected by the user, *N* the number of images contained in **C** and *M* the number of labels known for **C**. Parameters *M* and *N* were evaluated independently by holding the parameter not under consideration constant. The defaults for the parameter not under consideration were *n* = 1.0 and *m* = 0.0, as already mentioned when *n* = 1.0 and *m* = 0.0 the distance metrics 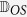 and 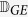 are equivalent. The parameters *M* and *N* were also evaluated together to explore the synergistic effects of *M* and *N* on image retrieval.

#### Effect of Known Label Size (*M*)

**Figure 7:**
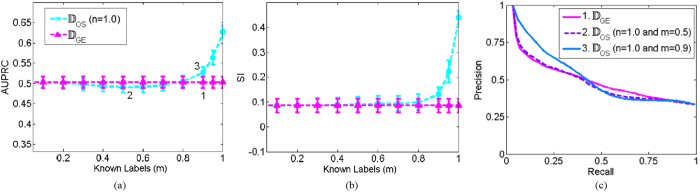
Effects of increasing the known labels (*M*) on the prostate histopatholgy database for (**a**) AUPRC and (**b**) SI in the low dimensional space obtained via OSE-SSL. The *X* axis reflects increasing *m*, defined as the size of the known labels (*M*) as a function of the percentage of the training set size (*N*). The pink line corresponds to the baseline case of *m* = 0.0. (**c**) Three example precision-recall curves for the AUPRC values indicated by the numbers 1, 2, and 3 in (**a**).

We hypothesized that adding label information via SSL would improve the ability of the low dimensional embedding space to distinguish between images belonging to different classes. Figure 7 displays the SI and AUPRC values of the baseline case of no labels (pink) and SSL by varying the number of known labels (light blue). Adding label information improved SI and AUPRC for large *M*.

#### Effect of Database Size (*N*)

**Figure 8:**
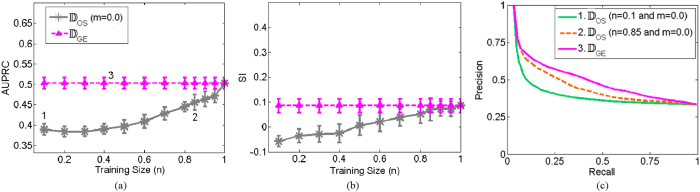
Effects of increasing the training set size (*N*) on the prostate histopatholgy database for (**a**) AUPRC and (**b**) SI in the low dimensional space obtained via OSE-SSL. The *X* axis reflects increasing *n* defined as the size of the training set (*N*) as a function of the percentage of the total dataset size (*N*_*all*_). The pink line corresponds to the baseline case of *n* = 1.0. (**c**) Three example precision-recall curves for the AUPRC values indicated by the numbers 1, 2, and 3 in (**a**).

We hypothesized for OSE-SSL small *N* would be unable to uncover the underlying structure in the database and result in embeddings which are less than optimal. As shown in Fig. 8, for *n* < 0.9 OSE was unable to accurately extrapolate embeddings. However, for *n* ≥ 0.9 there are no statistically significant differences (p-value > 0.05) between embeddings found via OSE and recomputing the EVD of the similarity matrix (i.e. embeddings found via GE).

#### Relationship between Database Size (*N*) and Known Label Size (*M*)

**Figure 9:**
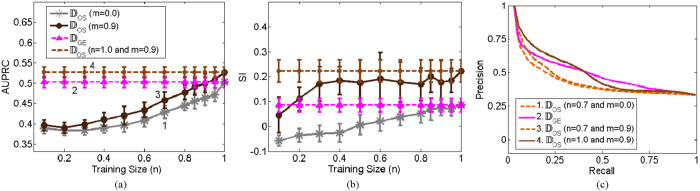
Effects of increasing the training set size (*N*) in conjunction with the known label (*M*) on the prostate histopatholgy database for (**a**) AUPRC and (**b**) SI in the low dimensional space obtained via OSE-SSL. The *X* axis reflects increasing the size of the training set (*N*) as a function of the percentage of the total dataset size. Different lines (0 and 0.9 are shown) reflect increasing the size of known labels as a function of the training set size. (**c**) Four example precision-recall curves for the AUPRC values indicated by the numbers 1, 2, 3, and 4 in (**a**).

The relationships between the SSL and OSE components of the OSE-SSL were evaluated. Increasing the known labels (*M*) necessitates a concomitant increase in database size (*N*) to appropriately model the embedding space. This trend is shown in Fig. 9 where for *m* = 0.0 a training set size of *n* = 0.9 is able to appropriately extrapolate embeddings. However, when *m* = 0.85 a training set size of *n* = 1.0 is required to appropriately extrapolate embeddings (i.e. GE must be utilized to learn the embeddings). In this database, *N* is not sufficiently high to capture the underlying structure if *M* is increased. Despite not having a large enough *N* to capture the underlying image structure increasing *M* does result in better AUPRC and SI measures even for small *N*.

### Experiment 3: Computational Time

**Figure 10:**
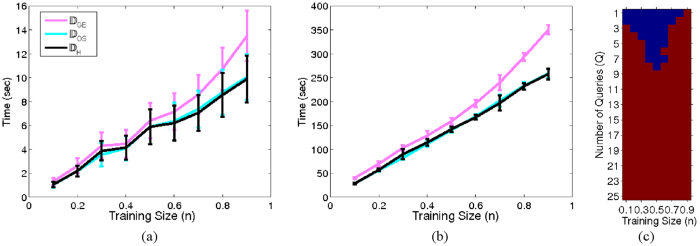
Time to retrieve database images for a query images using three distance metrics: 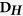 (H), 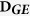 (GE), and 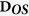 (OSE-SSL). The effects of training set size (*N*) and number of query images (*Q*) were evaluated. Retrieval time for (a) *Q* = 1 and (b) *Q* = 25 are shown, note the different y-axis scaling to better highlight the difference between the compared algorithms. 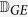 typically required more time to perform retrievals than either 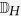 or 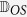. (c) Visual representation of when retrieval time difference for 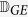 and 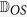 are statistically significant (p < 0.01, red) or not (p > 0.01, blue).

In this experiment we evaluated the time to retrieve images used the three distance metrics: 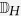, 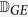, and 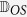 to retrieve relevant images for the prostate histopathology dataset using a range of training database sizes (*N*) and number of query images (*Q*). As shown in Fig. 10 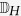 and 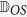 are able to retrieve images most similar to a query in approximately the same amount of time while 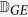 requires more time to perform an equivalent retrieval. Figure 10(c) displays under what conditions the time increases in retrieval for 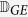 are statistically significant (red). For larger *N* and larger *Q*, 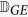 takes a statistically significant amount of time longer, the higher the values for *N* and *Q* the more pronounced this effect is. The increase in time for 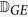 is due to two factors (a) 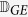 requires more pairwise comparisons between 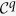 and the images contained in **C** and (b) 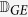 requires a computationally expensive EVD to compute *y*^*q*^, the low dimensional embedding for the query image.

## Discussion

In this paper we have presented a novel combination out-of-sample extrapolation with semi-supervised manifold learning (OSE-SSL) that first refines relationships between images in the low dimensional embedding space according to semantic information via SSL and then utilizes OSE to project never before seen images into the low dimensional space learned via SSL. We have demonstrated the application of OSE-SSL for content-based image retrieval (CBIR) of prostate histpathology. Image similarity within our CBIR framework is defined using Explicit Shape Descriptors (ESDs), ESDs were previously developed by our group to classify Gleason grade on prostate histopathology according to gland morphology^8^. In this work we leverage the accurate ESDs to determine similarity between images, and then apply the OSE-SSL algorithm to retrieve images which are most similar in a computationally efficient manner.

CBIR for histopathology, has as histopathology images require many image descriptors to accurately describe the large amounts of complex data present. Retrieval directly within the high dimensional feature space for histopathology images is difficult, as demonstrated by the relatively poor retrieval rates in the original high dimensional feature space 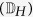 and using linear DR approaches 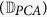.

Manifold learning (ML) can be leveraged to find a low dimensional representation where image similarity calculation and retrieval can be performed accurately and efficiently. In this paper we demonstrated that ML is able to retrieve histopathology images accurately, which has very limited previous work^6^,^7^

Our OSE-SSL CBIR algorithm was evaluated for a prostate histopathology database containing 888 glands. OSE-SSL outperformed image retrieval in the high dimensional space as well as in a low dimension space found by Principal Component Analysis (PCA) (as described in Experiment 1). We demonstrated that OSE-SSL was able to accurately retrieve images utilized a low dimensional embedding space found via SSL on a training database that was smaller compared to the full dataset. For the prostate histopathology dataset *M* = 0.85 of the dataset, or 754 images, was required to achieve retrieval rates comparable to those achieved by performing an EVD for each new query image. Finally, incorporating known label information was able to improve retrieval rates.

The current work is limited in that CBIR was performed on a per patch basis, where multiple patches are defined over a single slide. However, pathologists typically utilize the whole slide to determine Gleason grade pattern. Additionally, in this work we have leveraged only gland morphology to determine similarity between image patches. However, pathologists typically evaluate Gleason grade using the morphology and arrangement of glands and nuclei^2^. Future work will involve incorporating our gland based retrieval into a whole slide similarity metric, which will be capable of retrieving whole slides which contain similar image characteristics, likely including measures of nuclei arrangement^43^ and nuclei morphology^44^.

The current work is also limited by the fact that all 58 patients had prostate tissue biopsy cores acquired at a single institution. Therefore, the dataset used in this work may be more homogeneous, in terms of tissue staining and digitization of the slides, compared to a dataset of prostate histopathology images acquired across several institutions. While these differences between institutions will likely affect pre-processing steps such as automated segmentation, in this work we have limited the effects of a homogeneous dataset by relying on manual segmentation. The variability in gland morphology is independent of institution, as gland morphology is a function of disease grade. Future work will evaluate the presented methodology on a larger patient cohort acquired across institutions.

Additionally, the current work only evaluated morphologic features (ESDs) of glands present on prostate histopathology. Previous work has shown that texture^6^,^7^ and nuclear architecture^6^,^7^,^11^ are also able to provide accurate image retrieval of prostate histopathology. The OSE-SSL algorithm is not limited to ESDs, hence, alternative dissimilarity measures that combine ESDs with other features derived from the prostate histopathology images can be implemented within our CBIR framework. Future work will evaluate the dissimilarity measures that combine multiple image features.

## Additional Information

**How to cite this article**: Sparks, R. and Madabhushi, A. Out-of-Sample Extrapolation utilizing Semi-Supervised Manifold Learning (OSE-SSL): Content Based Image Retrieval for Histopathology Images. *Sci. Rep.* **6**, 27306; doi: 10.1038/srep27306 (2016).

## Figures and Tables

**Figure 1:**
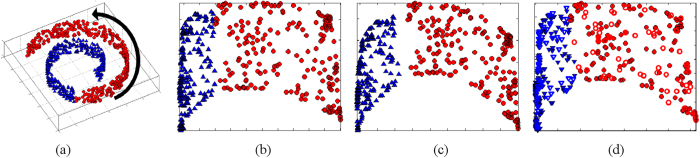
(**a**) 3*D* Swiss Roll dataset comprising 2000 samples divide in two classes (red, blue). The arrow displays the direction of greatest variance along the manifold. (**b**) 2*D* low dimensional embedding space found via Graph Embedding (GE). Note that the two classes cluster on different regions of the low dimensional embedding space. (**c**) 2*D* low dimensional embedding space found via semi-supervised GE (SSGE). Note that the two classes are more separated than for GE. (**d**) 2*D* low dimensional embedding space found via GE (closed points) and OSE (open points).

**Table 1:** Acronyms used throughout this paper in order of appearance.

Acronym	Description	Acronym	Description
ESDs	Explicit Shape Descriptors^8^	LPP	Locality Preserving Projections^28^
ML	Manifold learning	OSE	Out-of-sample extrapolation
CBIR	Content-based image retrieval	MDS	Multi-dimensional scaling
DR	Dimensionality reduction	ROI	Region of interest
PCA	Principal Component Analysis	H & E	Hemotoxylin and Eosin
SSL	Semi-supervised learning	BE	Benign
LDA	Linear Discriminant Analysis	G3	primary Gleason grade 3
GE	Graph Embedding^23^	G4	primary Gleason grade 4
SSGE	Semi-supervised Graph Embedding	SI	Silhouette index^41^
EVD	Eigenvalue decomposition	AUPRC	Area under the precision recall curve

**Table 2:** Notation used throughout this paper.

Symbol	Definition	Symbol	Definition
**C**	Image database	*N*	Number of images in **C**
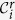	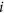 th image in **C**	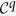	User-selected query image
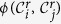	Dissimilarity function between 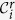 and 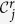	**y**^*r*^	Embeddings in the low dimensional space 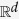
**L**	Image label information	*A*	Dissimilarity matrix for **C**
*W*	Similarity matrix for **C**	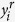	Embedding location for 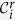
*y*^*q*^	Embedding location for 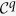	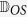	Distance metric in 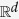

**Table 3:** Evaluation measures to compare CBIR systems.

Measure	Description
Silhouette Index (SI)	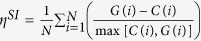 where 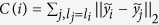 and 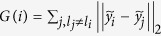
Area Under the Precision Recall Curve (AUPRC)	Area generated by plotting *p*(*α*) versus *r*(*α*) where 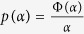 and 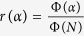 . Φ(*α*) denotes the number of relevant objects in the closest *α* points. *p*(*α*) and *r*(*α*) are evaluated for *α* ∈ {1, …, *N*} to obtain the full precision recall curve.

**Table 4:** Comparative distance metrics utilized to define alternative image similarity measures.

Distance Metric	Description	Supervision Type
High dimension 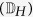	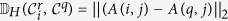 .	Unsupervised
Principal Component Analysis 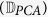	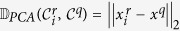 , where 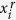 , *x*^*q*^ are obtained by projecting *A*(*i*, ·) into a lower dimensional coordinate space defined by the top *d* principal components obtained from PCA.	Unsupervised
Locality Preserving Projections 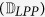	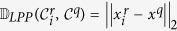 , where 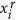 , *x*^*q*^ is obtained by projecting *A*(*i*, ·) into a lower dimensional coordinate space defined by the top *d* dimensions obtained from LPP^28^.	Unsupervised
Graph Embedding 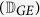	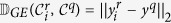 , where 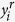 , *y*^*q*^ are obtained by applying GE to *A* as described in Equation 2. 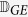 is equivalent to 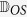 with *n* = 1 and *m* = 0.	Unsupervised
Kernel-based Hashing 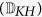	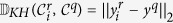 , where 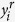 , *y*^*q*^ are a set of 32-bit hash code. Hash codes are obtained by learning a mapping from a kernel space, describe by *κ*(*A*(*i, j*) − *A*(*q, j*)) where *κ*(·) is a kernel function, onto a low dimensional hash space as described in^42^.	Semi-supervised

**Table 5:** AUPRC and SI values for Experiment 1.

Distance Metric	Evaluation Measure				
	Dimension	AUPRC	p-value	SI	p-value
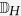	–	0.42 ± 0.01	9.8E – 23	−0.06 ± 0.02	2.0E – 14
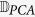	25	0.44 ± 0.01	5.5E – 13	−0.10 ± 0.02	2.0E – 15
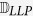	7	0.43 ± 0.02	3.8E – 7	0.03 ± 0.07	2.6E – 7
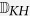	–	0.41 ± 0.01	2.3E – 7	0.02 ± 0.12	3.2E – 10
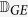	7, *σ* = 100	0.48 ± 0.01	–	0.08 ± 0.03	–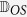	7, *σ* = 100	**0.53** ± **0.02**	6.1E – 3	**0.14** ± **0.12**	2.8E – 6

Values for the best performing metric are bolded. p-values used a paired two-sided Student’s t-test to evaluate whether the distance metric 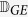 outperformed a comparative distance metric (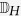, 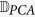, or 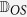), the null hypothesis being the two metrics are equivalent.
